# Comparative transcriptomics identifies the key *in planta*-expressed genes of *Fusarium graminearum* during infection of wheat varieties

**DOI:** 10.3389/fgene.2023.1166832

**Published:** 2023-04-18

**Authors:** Qiang Tu, Lirong Wang, Qi An, Jie Shuai, Xiaobo Xia, Yifan Dong, Xu Zhang, Gang Li, Yi He

**Affiliations:** ^1^ CIMMYT-JAAS Joint Center for Wheat Diseases, The Research Center of Wheat Scab, Jiangsu Academy of Agricultural Sciences, Nanjing, China; ^2^ School of Life Sciences and Engineering, Wheat Research Institute, Southwest University of Science and Technology, Mianyang, Sichuan, China; ^3^ Department of Plant Pathology, College of Plant Protection, Nanjing Agricultural University, Nanjing, China; ^4^ Jiangsu Co-Innovation Center for Modern Production Technology of Grain Crops, Yangzhou University, Yangzhou, China

**Keywords:** transcriptome, gene expression, Fusarium head blight, resistance, susceptibility, F. graminearum, wheat

## Abstract

Fusarium head blight (FHB), caused mainly by the fungus *Fusarium graminearum*, is one of the most devastating diseases in wheat, which reduces the yield and quality of grain. *Fusarium graminearum* infection of wheat cells triggers dynamic changes of gene expression in both *F. graminearum* and wheat, leading to molecular interactions between pathogen and host. The wheat plant in turn activates immune signaling or host defense pathways against FHB. However, the mechanisms by which *F. graminearum* infects wheat varieties with different levels of host resistance are largely limited. In this study, we conducted a comparative analysis of the *F. graminearum* transcriptome *in planta* during the infection of susceptible and resistant wheat varieties at three timepoints. A total of 6,106 *F. graminearum* genes including those functioning in cell wall degradation, synthesis of secondary metabolites, virulence, and pathogenicity were identified during the infection of different hosts, which were regulated by hosts with different genetic backgrounds. Genes enriched with metabolism of host cell wall components and defense response processes were specifically dynamic during the infection with different hosts. Our study also identified *F. graminearum* genes that were specifically suppressed by signals derived from the resistant plant host. These genes may represent direct targets of the plant defense against infection by this fungus. Briefly, we generated databases of in planta-expressed genes of *F. graminearum* during infection of two different FHB resistance level wheat varieties, highlighted their dynamic expression patterns and functions of virulence, invasion, defense response, metabolism, and effector signaling, providing valuable insight into the interactions between *F. graminearum* and susceptible/resistant wheat varieties.

## Introduction

Wheat is a major cereal crop that feeds more than 35% of the global population (Zhu et al., 2021). However, its productivity and quality are drastically limited by many fungus-associated diseases such as Fusarium head blight (FHB). FHB is mainly caused by the pathogen *Fusarium graminearum*, a hemibiotrophic and plant pathogenic ascomycete fungus causing diseases in many cereal crops (Khan et al., 2020). The average global wheat yield loss caused by FHB is reported to be second only to that caused by wheat leaf rust (Savary et al., 2019). Serious FHB epidemics usually occur every 4, 5 years in wheat-growing areas worldwide (Figueroa et al., 2018). Infection with *F. graminearum* leads to shriveled, discolored kernels or total failure of kernel development, which affects crop yield and quality (Trail, 2009). Moreover, *F. graminearum*-secreted mycotoxins, especially deoxynivalenol (DON), strongly adversely affect mammal health (Scudamore, 2008). To ensure the safety of food, most countries have strict limits on the level of it in grain, food, and feed (Ji et al., 2014; Bianchini et al., 2015).

In the early stage of infection, *F. graminearum* germ tubes produced by spores extend and branch on the surface of host cells, and then develop into hyphal networks under high-moisture and high-temperature conditions (Kang et al., 2004). The extended fungal hyphae penetrates host tissues through wounds or natural floret openings such as the inner surfaces of glume, lemma, palea, and the upper part of the ovary (Lewandowski et al., 2006). Infection cushions are usually formed during the process of infection (Boenisch and Schafer, 2011), which are agglomerations of fungal hyphae secreting various hydrolyzing enzymes, such as cutinases, pectinases, cellulases, xylanases, and lipases. These in turn degrade components of the epidermal plant cuticle and the plant cell wall (Cuomo et al., 2007; Walter et al., 2010). Although no visible symptoms of infection are observed at this stage, many genes related to pathogenicity and virulence are known to be expressed, which mostly encode fungal intracellular proteins, including transcription factors, protein kinases, phosphatases, and primary metabolism enzymes (Son et al., 2011; Yun et al., 2015). Some genes involved in secondary metabolite biosynthesis (SMB) have a strong toxic effect on wheat (Sieber et al., 2014; Macheleidt et al., 2016). These genes are usually organized into clusters, such as the fg3-54 cluster, with many of them encoding classic SMB-related enzymes such as non-ribosomal peptide synthetases (NRPS), polyketide synthases, and terpene cyclases (Jia et al., 2019). The trichothecene toxin DON is particularly crucial for *F. graminearum* infection. DON allows the invading fungus to spread through the rachis from infected to adjacent spikelet, and suppresses cell wall thickening at the rachis node as a translational inhibitor (Boenisch and Schafer, 2011). *TRI5* (*FGSG_03537*) from *F. graminearum* is essential for DON biosynthesis, as loss-of-function mutations have been shown to result in a lack of DON production and reduced virulence (Jansen et al., 2005). Many genes related to pathogenicity, virulence, or growth in the early infection period have also been reported in previous studies. An example of this is *NPS6*, which encodes a non-ribosomal peptide synthetase involved in virulence and hypersensitivity to H_2_O_2_ (Oide et al., 2006). Another example is *FGL1* (*FGSG_05906*), which encodes a lipase releasing free fatty acids to inhibit innate immunity-related callose (Blumke et al., 2014).

When plants are infected by pathogens, a large number of pattern recognition receptors on the surface of host cells, including receptor-like proteins and receptor-like kinases, can recognize pathogen-associated molecular patterns (PAMPs), which induces plant PAMP-triggered immunity (PTI) (Zhou and Zhang, 2020). To overcome the host’s PTI, fungi secrete a range of molecules that contribute to their infection, called effectors, which play important roles in pathogen–host interactions (Gorash et al., 2021). Plants in turn activate effector-triggered immunity upon sensing effectors through the hypersensitive cell death (HR) response (Gorash et al., 2021). Instead, HR-induced cell death may provide nutrition for necrotrophic pathogens that feed on dead cells (Zhou and Zhang, 2020). *Fusarium graminearum* is a hemibiotroph combining certain features of both biotrophic and necrotrophic pathogens, which may possess a particularly complicated mechanism to penetrate and parasitize plant cells, making it difficult to control (Duba et al., 2018). Using a large number of genetic screenings, several varieties, such as Sumai3 and Wangshuibai, have been revealed to be highly resistant to FHB, which carries the *Fhb1* QTL (Lin et al., 2004; Cuthbert et al., 2006). The candidate gene *Fhb1* has recently been cloned from Sumai3 and Wangshuibai, which encodes a histidine-rich calcium-binding protein, but the resistance mechanism remains unknown (Li et al., 2019; Su et al., 2019). Owing to the complicated mechanism by which *F. graminearum* infects host cells, the interaction between pathogen and wheat during FHB is still largely limited.

Transcriptomic analysis is a highly sensitive and comprehensive strategy for evaluating gene expression. In recent years, transcriptomic analysis based on RNA sequencing has been widely used to dissect key genes and underlying mechanisms involved in host–*F. graminearum* interactions (Kazan and Gardiner, 2018). Most studies have focused on comparing the expression of genes during the infection of a single plant host, including *F. graminearum* genes and plant host genes, or comparing the expression of host genes between different plant hosts (Boedi et al., 2016; Chetouhi et al., 2016; Puri et al., 2016; Mentges et al., 2020; Yang et al., 2022; Yin et al., 2022). However, the mechanistic interplay between *F. graminearum* and different wheat varieties during FHB infection, especially for susceptible and resistant varieties, needs further analysis. In this study, a comparative transcriptomic analysis was conducted to examine *in planta* gene expression of *F. graminearum* in susceptible and resistant wheat heads at different stages of infection. Overall, 6,106 *F. graminearum* genes expressed in susceptible and resistant wheat varieties were identified. Our study showed that the expression pattern of the *F. graminearum* genes was regulated by hosts with different genetic backgrounds and provided valuable insights into the interactions between *F. graminearum* and susceptible/resistant wheat varieties. This work also provides potential targets for the pharmaceutical control of *FHB*.

## Materials and methods

### Plant materials and growth conditions

Seeds of wheat varieties Fielder (FHB-susceptible) and Sumai3 (FHB-resistant) were placed on sterile filter paper, which was then placed inside a sterile Petri dish with 5 mL of sterile water. All seeds were incubated in the dark for 5 days at 4°C before transplanting into 4 L pots in a greenhouse under optimal conditions to allow tillering and synchronized flowering. An artificial watering system was installed, and the daily photoperiod was set at 16 h of daylight at a temperature of 20°C and 8 h of darkness at 18°C.

### *Fusarium graminearum* inoculation

Spikes showing the same ontogeny on the same day (midanthesis) were used for *F. graminearum* inoculation. Ten microliters of *F. graminearum* strain Fg1312 at a concentration of 10^5^ spores per ml was injected into one floret at intermediate positions of spikes and covered with a plastic bag for 3 days to ensure moisture retention. Plants were inoculated with water as a control (mock). The inoculated spikelets were collected at three infection timepoints (12, 24, and 36 h after inoculation (HAI)), and stored at −80°C. Three biological replicates were performed.

### RNA isolation and RNA-Seq

Spikelets from 12, 24, and 36 HAI were used for RNA-Seq. Total RNA was extracted using TRIzol reagent from flash-frozen spikelets, in accordance with the manufacturer’s instructions (Invitrogen), and were then sent to Qing Lian Bio (Beijing, China) for further purification and sequencing. Library preparation was performed using 1 μg of high-integrity total RNA (RIN >8) with the NEBNext^®^ Ultra™ RNA Library Prep Kit (NEB), followed by sequencing using Illumina HiSeq in paired-end mode with a read length of 150 bp. The quality of raw sequencing reads for all samples was examined using FastQC (http://www.bioinformatics.bbsrc.ac.uk/projects/fastqc/). Raw reads were filtered by removing adapters and clean reads were aligned to the PH-1 RefSeq assembly using HISAT2 (version 2.1.0). The expected number of fragments per kilobase of transcript sequence per million base pairs sequenced (FPKM) of each gene was calculated based on the length of the gene and read count mapped to the gene.

### Identification of expressed genes and differentially expressed genes

Identification of expressed genes and differentially expressed genes was performed using the FPKM value. The original FPKM was statistically analyzed using SPSS software, including principal component analysis and correlation analysis among three biological repeats. Genes with log2 (fold change) ≥1 and *P*_adj_ < 0.05 between fungus-inoculated samples and mock-inoculated samples were considered to be expressed.

Genes with |log2 (foldchange)| ≥1 and *P*_adj_ < 0.05 between different timepoints were identified as being differentially expressed. Bar diagrams were created with GraphPad Prism 9.0.0. Venn diagrams were created at https://www.bioinformatics.com.cn. All bar and venn diagrams were modified using Adobe Illustrator 2020 software (https://www.adobe.com/cn/products/illustrator.html). Heatmaps were produced using TBtools software (Chen et al., 2020).

### Bioinformatics and statistical analysis

The DEGs were subjected to Gene Ontology (GO) functional enrichment analysis and Kyoto Encyclopedia of Genes and Genomes (KEGG) pathway enrichment analysis using an online database (https://fungidb.org/fungidb/app). All user-defined parameters were set to the program’s default settings. GO terms and KEGG pathways with a *p*-value of <0.05 were considered to be significantly enriched. The interactions between DEG-encoded proteins were analyzed using the online database STRING (https://cn.string-db.org/). The input gene set was set as the DEGs, and the species was set as *F. graminearum* PH-1. The predicted PPI network was built using Cytoscape software (https://cytoscape.org/). The CytoNCA module in Cytoscape was used to analyze the network topology properties of the nodes. Through ranking the score of each node, the important nodes of the PPI network were obtained. The predicted DEGs were also subjected to GO and KEGG enrichment analyses. The enriched important DEGs were displayed in different colors in the network. Putative effector proteins within the refined secretome were predicted using EffectorP 3.0 (Sperschneider and Dodds, 2022). Phenotypic data of all published studies on single-gene deletion of *F. graminearum* were retrieved from PHI-base (Urban et al., 2020).

### qRT-PCR analysis

Differentially expressed genes (DEGs) were randomly selected and analyzed by quantitative reverse transcription PCR (qRT-PCR) to verify the reliability of the sequencing data. The first-strand cDNAs were synthesized using the PrimeScript first strand cDNA Synthesis Kit (Takara Bio, Dalian, China). qRT-PCR was performed using a Roche thermal cycler 96 with the SYBR Green reagent (Takara Bio, Dalian, China), including 2 μL of cDNA, 5 μL of TB Green^®^ Premix Ex Taq™ II reagent and 1 μL of each primer (10 mM) in a final volume of 10 μL reaction solution. The PCR procedure was performed as described previously (He et al., 2020), which started at 95°C for 30 s, followed by 45 cycles of 95°C for 5 s, 60°C for 20 s, and 72°C for 10 s. Melting curve analysis included 95°C for 10 s, 65°C for 15 s, and heating to 95 °C at a rate of 0.1 °C/s with continuous readings of fluorescence for each amplification. The *F. graminearum* gene *FgEF1A* was used as the internal control. The primers used in this study are listed in Supplementary Table S1.

## Results

### *In planta* identification of expressed *Fusarium graminearum* genes during infection

**FIGURE 1:**
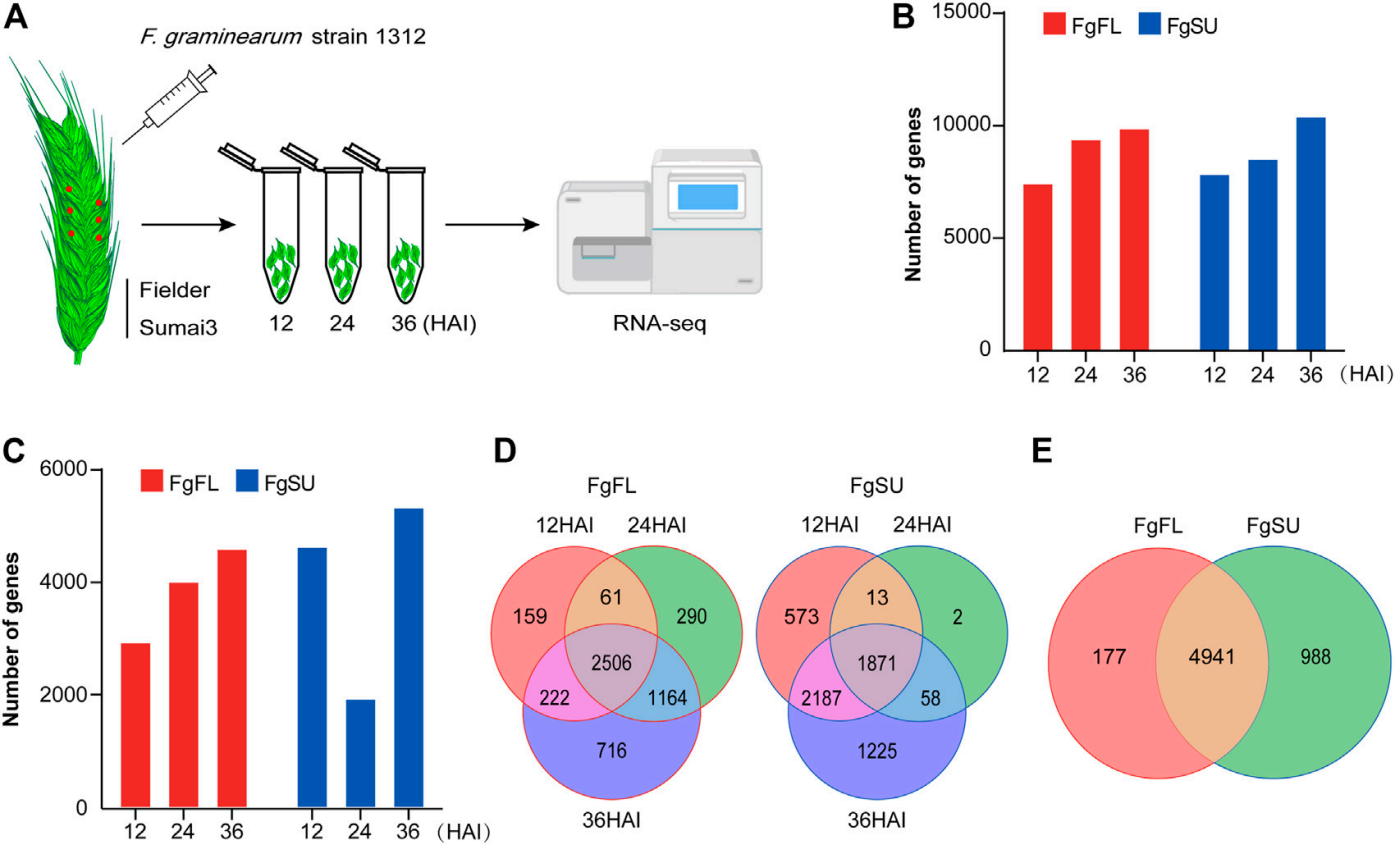
Inoculation procedure and transcriptomics of *Fusarium graminearum in planta*. **(A)** Schematic workflow of *Fusarium graminearum* transcriptomic analysis *in planta*. Briefly, the FHB-susceptible wheat variety Fielder and FHB-resistant wheat variety Sumai3 were cultivated to midanthesis under standard conditions. Then, six spikelets on three spikes of each variety were point-inoculated using *Fusarium graminearum* strain Fg1312 inoculum or water (Mock). At 12, 24, and 36 HAI, these inoculated materials were harvested separately and used for RNA-Seq. The reads generated by RNA-Seq *in planta* were then mapped to the *Fusarium graminearum* genome. **(B)** Number of genes mapped to the *Fusarium graminearum* PH-1 genome. **(C)** Number of *in planta*-expressed genes identified at three timepoints after inoculation. Log2 (foldchange) ≥1 and *P*_adj_ < 0.05 between *Fusarium graminearum*-inoculated samples and mock-inoculated samples were used as the criteria to identify *Fusarium graminearum* genes expressed *in planta*, which can eliminate the interference of the wheat genome. **(D)** Dynamic distribution of genes expressed in the different varieties. **(E)** Venn diagrams of all genes expressed in the different varieties. Most genes (∼81%) were expressed in both varieties, while a few genes were specifically expressed in Fielder (∼3%) or Sumai3 (∼16%). FgFL, F. graminearum genes expressed in Fielder; FgSU, F. graminearum genes expressed in Sumai3.

The first 36 h after infection (HAI) was the key time for *F. graminearum* to colonize wheat, as the macroconidia germinated with germ tubes within 12 HAI, and expanded on the surfaces of the lemma and ovary with invasive hyphae which had already secreted trichothecene toxins within 36 HAI (Kang et al., 2004). To get a comprehensive view of the *in planta* gene expression of *F. graminearum* in susceptible and resistant wheat heads within 36 HAI, we profiled the transcriptome analysis using RNA-Seq in wheat spikes of susceptible and resistant varieties, Fielder and Sumai3, respectively (Figure 1A). In contrast to most *in vitro* global gene expression assays of *F. graminearum*, we used the plant tissues for infection and subsequent RNA-Seq, while the reads were mapped to the *F. graminearum* genome. Thus, the results present highly reliable genes of *F. graminearum* expressed *in planta* during the infection period. A total of 11,314 genes were detected at 12, 24, and 36 HAI (Figure 1B; Supplementary Table S2). To confirm the reproducibility of the sequencing data, correlation analysis was conducted among the three biological replicates, and all showed a high correlation (Supplementary Figure S1A; Supplementary Table S2). In principal component analysis, samples undergoing the same treatment were found to be clustered together, while susceptible variety Fielder showed a wider variation than resistant variety Sumai3, which explained 25.43% of the variation of PC1 (Supplementary Figure S1B; Supplementary Table S2).

To avoid the wheat genome interference from wheat, genes with log_2_ (foldchange) ≥1 and *P*_adj_ < 0.05 between *F. graminearum*-inoculated samples and mock-inoculated samples were considered to be *in planta*-expressed (Figure 1C; Supplementary Table S2). In Fielder, the number of expressed genes increased with increasing infection duration, while in Sumai3, the number decreased significantly at 24 HAI (Figure 1C). Most genes were found to be expressed at two or three timepoints after inoculation, while a few were detected at only a single timepoint (Figure 1D). In total, 11,314 genes were detected in at least one timepoint, and 6,106 were identified as expressed in *F. graminearum* with high confidence based on foldchange and *P*_adj_ criteria, of which 5,118 were identified in Fielder and 5,929 in Sumai3, with 80.9% (4,941 genes) of them being expressed in both varieties (Figure 1E).

### Dynamic distribution of *Fusarium graminearum in planta* DEGs in different wheat varieties

**FIGURE 2:**
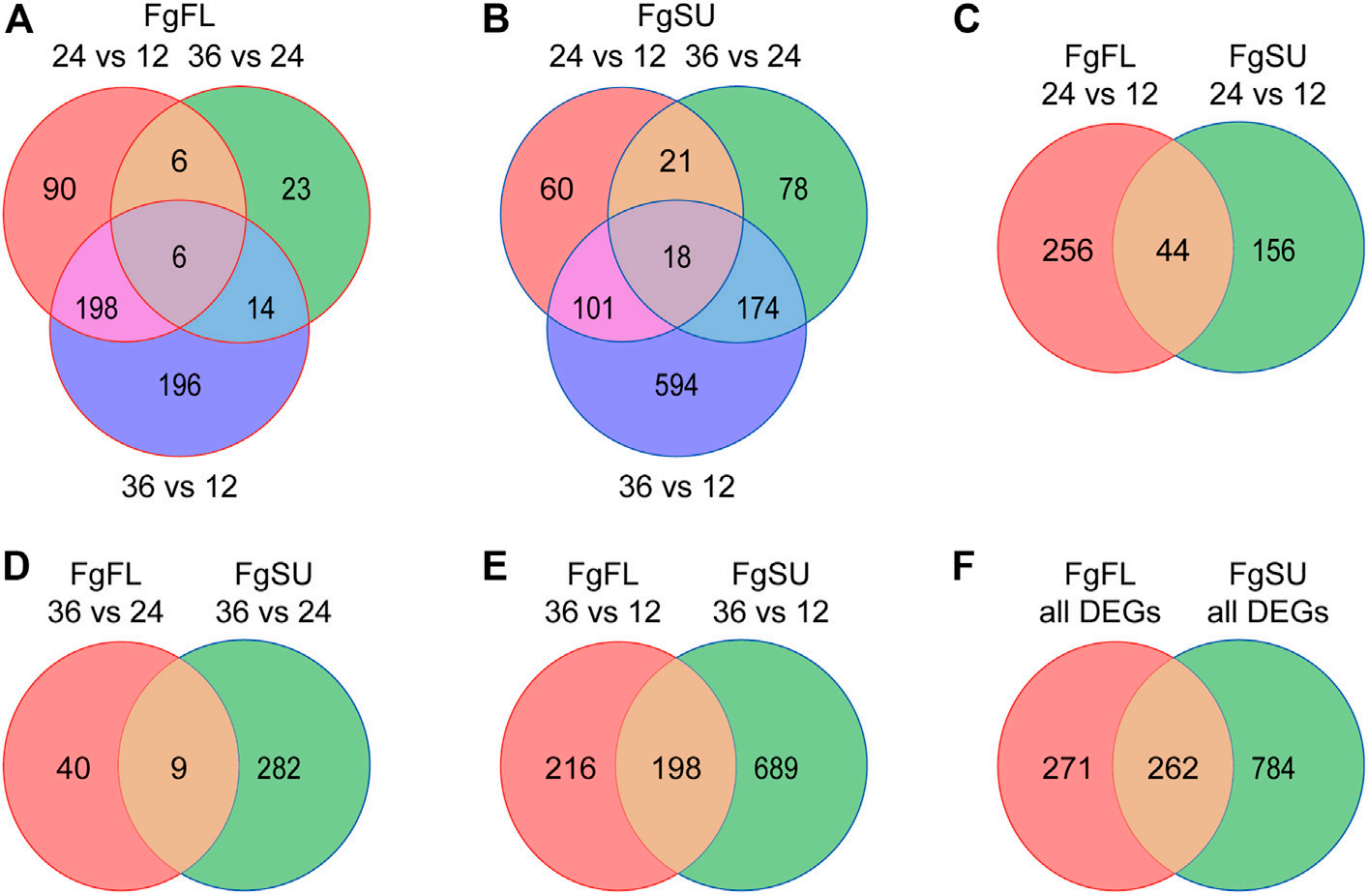
Dynamic distribution of *Fusarium graminearum* DEGs in hosts. **(A)** and **(B)** Venn diagrams showing the dynamic distribution of differentially expressed *Fusarium graminearum* genes during infection of the wheat varieties Fielder **(A)** and Sumai3 **(B)**. All of the DEGs were identified from the three comparisons. **(C–E)**. Venn diagrams showing the distribution of *Fusarium graminearum* genes differentially expressed between Fielder and Sumai3. **(F)** Venn diagram of the distribution of all differentially expressed *Fusarium graminearum* genes identified in Fielder and Sumai3 within 36 HAI.

To further catalog the genes with significant changes among all expressed genes, DEG analysis was conducted between different timepoints after inoculation (Supplementary Table S2). Upon comparisons of 24 HAI vs. 12 HAI, 36 HAI vs. 24 HAI, and 36 HAI vs. 12 HAI, 300, 49, and 414 genes were identified as DEGs in the three comparisons, respectively, and a total of 533 DEGs were identified in Fielder (Figure 2A; Supplementary Table S2). In Sumai3, 1,046 DEGs were identified, and the numbers of DEGs in the three comparisons were 200, 291, and 887, respectively (Figure 2B; Supplementary Table S2). We further analyzed the distribution of DEGs between resistant and susceptible varieties, and found that 456, 331, and 1,103 DEGs were identified in the three comparisons, respectively (Figures 2C–E). In the early stage of infection, more DEGs were detected in Fielder, but with an increasing duration of infection, more were detected in Sumai3. Most of the DEGs in Fielder or Sumai3 were identified in the comparison of 36 HAI vs. 12 HAI (Figure 2E). In summary, a total of 1,317 DEGs were identified in Fielder and Sumai3, but many DEGs were Sumai3-specific (Figure 2F; Supplementary Table S2). These *in planta* DEGs caused by *F. graminearum* infection differed in their response timing and expression patterns, which likely depends on the pathogen’s interactions with susceptible and resistant hosts. Together, our data indicate that wheat varieties showing different abilities to resist FHB may influence *F. graminearum* infection through regulating the pathogen’s gene expression.

### Functional enrichment of *F. graminearum* DEGs reveals different *F. graminearum* infection mechanisms in susceptible and resistant hosts

**FIGURE 3:**
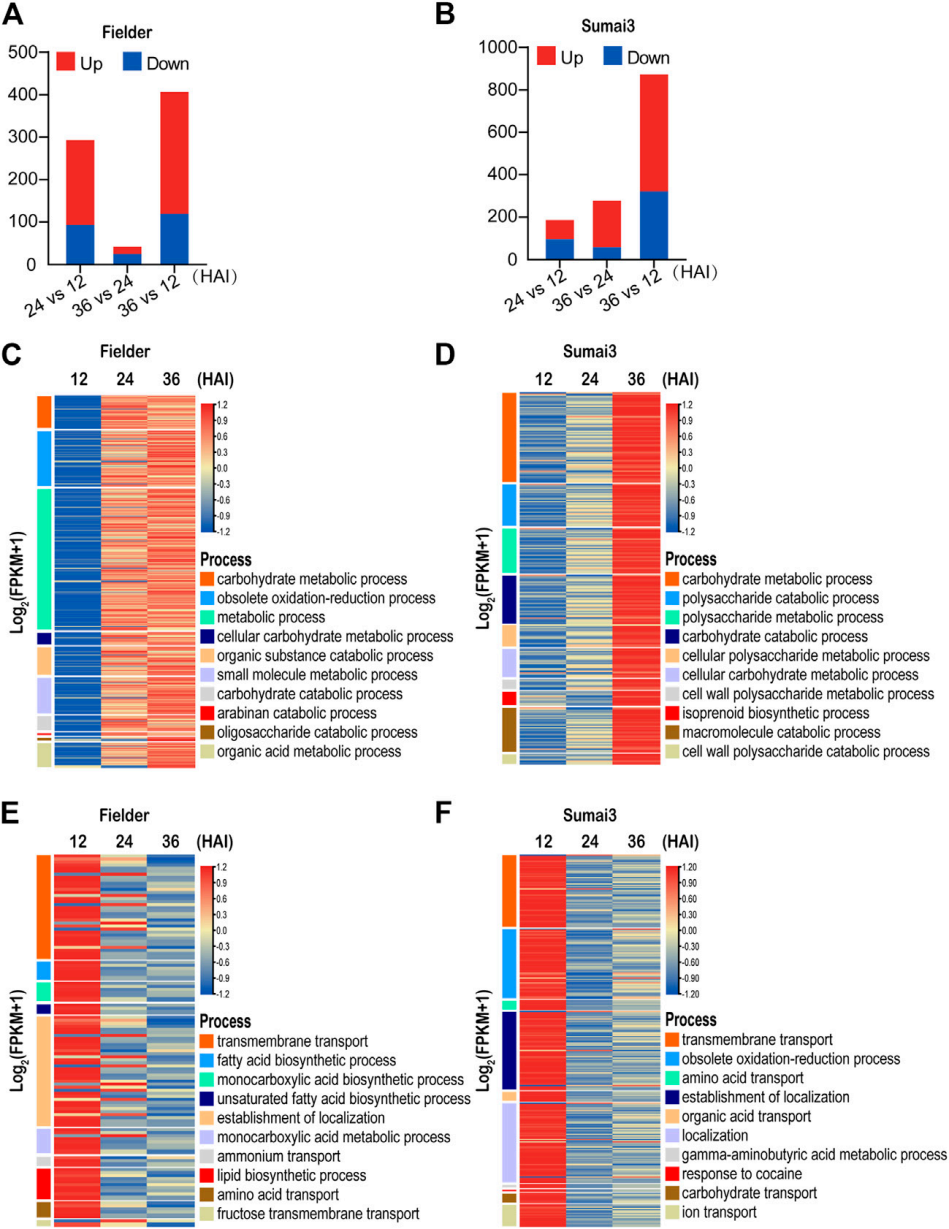
Temporal expression patterns and GO analysis of differentially expressed *Fusarium graminearum* genes in Fielder and Sumai3 **(A)** and **(B)** The numbers of significant DEGs in Fielder and Sumai3 within 36 HAI. Genes with log_2_ (foldchange) ≥1 and p_adj_ < 0.05 were identified as upregulated, while those with log_2_ (foldchange) ≤−1 and p_adj_ < 0.05 were identified as downregulated. **(C)** and **(D)** Heatmaps of significantly enriched GO categories associated with *Fusarium graminearum* genes upregulated in Fielder and Sumai3. **(E)** and **(F)** Heatmaps of significantly enriched GO categories associated with *Fusarium graminearum* genes downregulated in Fielder and Sumai3.

To obtain insights into the DEGs between susceptible and resistant hosts, we first divided all DEGs into two categories: upregulated upon infection [log_2_ (foldchange) ≥1, p_adj_ < 0.05)] and downregulated upon infection [log_2_ (foldchange) ≤ −1, p_adj_ < 0.05]. Among the 533 DEGs identified in Fielder, 372 were upregulated and 168 were downregulated upon infection in the different comparisons, and the identified changes in expression mainly occurred in the comparisons of 24 HAI vs. 12 HAI and 36 HAI vs. 12 HAI (Figure 3A; Supplementary Table S3). In contrast, among 1,046 DEGs identified in Sumai3, 683 were upregulated and 396 were downregulated, and a majority of the DEGs were identified in the comparison of 36 HAI vs. 12 HAI (Figure 3B; Supplementary Table S3). This contrasts with the changes of expression pattern observed in Fielder, which further indicates the different mechanisms of *F. graminearum* infection in susceptible and resistant varieties.

We then conducted GO enrichment analysis of the upregulated DEGs. In the process of infecting Fielder, we identified transcriptional response categories related to growth, reproduction, and host invasion as the most enriched processes for the upregulated genes (Supplementary Table S3). The 10 most significantly enriched GO terms were associated with basic catabolism, metabolism after inoculation, including carbohydrate metabolic process, and metabolic process (Figure 3C). The significantly enriched processes related to catabolism and metabolism without visible symptoms on wheat spikelets supported the biotrophic lifestyle of *F. graminearum* in the early period of infection (Gorash et al., 2021). There was also enrichment of DEGs associated with defense response processes and host invasion items such as obsolete oxidation–reduction and small-molecule metabolic processes (Figure 3C). Similar GO items in Sumai3 were found when compared to Fielder. Specifically, 136 and 140 significantly enriched biological process categories were identified in Fielder and Sumai3, respectively, including catabolism, metabolism, and defense response (Supplementary Table S3). The similarity of enriched categories suggested that there were no significant differences between susceptible and resistant varieties after infection in terms of growth and development, but there was a higher level of transcript abundance at 24 HAI in Fielder and 36 HAI in Sumai3 (Figures 3C, D). We hypothesize that this may underpin the resistance of Sumai3 to *F. graminearum* and was further supportive of a different temporal response between susceptible and resistant hosts.

To identify biological processes that were suppressed in each host over the time course of infection, we also conducted GO enrichment analysis of downregulated DEGs. As illustrated in Figure 3E, 168 DEGs were identified in Fielder, which were particularly associated with 101 biological process categories, while transmembrane transport, localization, as well as acid metabolism were significantly enriched terms (Figure 3E; Supplementary Table S3). Meanwhile, 396 downregulated DEGs and 84 enriched categories were identified in Sumai3. Among these, some processes were similar to those in Fielder, such as “localization” and “transport,” while some were involved in response to host immunity, such as “obsolete oxidation–reduction process,” “response to toxic substance,” and “response to fungicide” (Figure 3F; Supplementary Table S3). Taking the findings together, our analyses emphasized the functional differences of DEGs in susceptible and resistant hosts.

### Differential patterns of infection responses in Fielder and Sumai3

**FIGURE 4:**
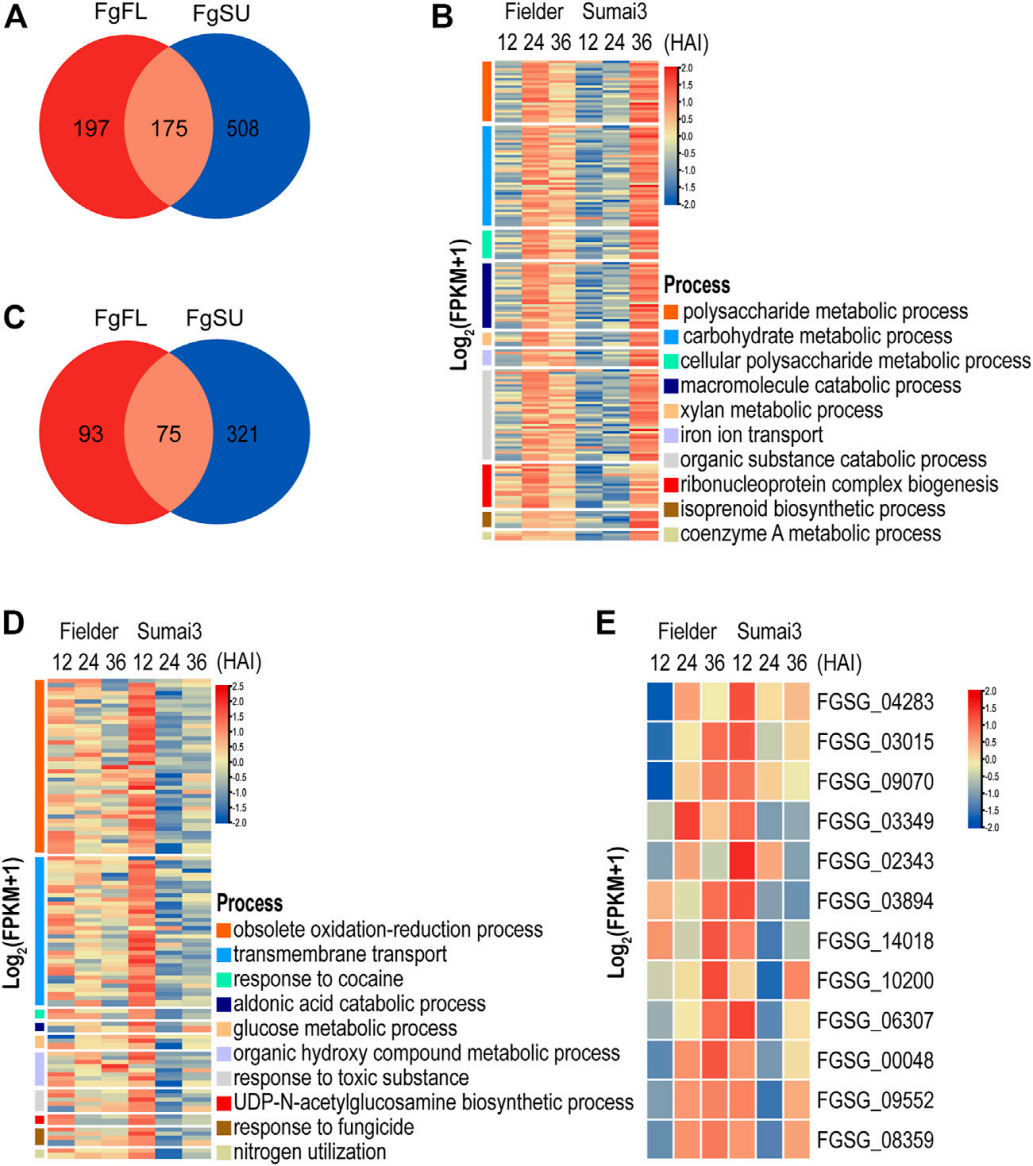
Temporal expression pattern and function analyses of DEGs that were specifically upregulated or downregulated in resistant host **(A)** Distribution of DEGs that were upregulated in different hosts. **(B)** GO terms significantly associated with DEGs specifically upregulated in resistant host. **(C)** Distribution of DEGs that were downregulated in different hosts. **(D)** GO terms significantly associated with DEGs specifically downregulated in resistant host. **(E)** Heatmap of DEGs that were upregulated in susceptible host but downregulated in resistant host.

To determine the difference of temporal expression pattern of the same DEGs between resistant and susceptible hosts, upregulated and downregulated DEGs were divided into three categories depending on whether they were Fielder-specific, Sumai3-specific, or their differential expression occurred in both hosts (Supplementary Table S4). As shown in Figure 4A, we identified 880 significantly upregulated DEGs, with 508 being specifically upregulated in Sumai3, while only 197 were specifically upregulated in Fielder. Meanwhile, 175 were upregulated in both hosts after infection (Figure 4A; Supplementary Table S4). To further explore the biological processes associated with the list of classified DEGs, GO enrichment analysis was conducted in six categories (Supplementary Table S4). DEGs that were specifically upregulated in Fielder were particularly associated with “obsolete oxidation–reduction process,” “cellular amino acid catabolic process,” and “glycine metabolic process” (Supplementary Table S4). DEGs that were upregulated in both hosts were particularly associated with “carbohydrate metabolic process,” “metabolic process,” and “cellular carbohydrate metabolic process” (Supplementary Table S4). However, DEGs that were upregulated and specific to Sumai3 were particularly associated with “polysaccharide catabolic process,” “carbohydrate catabolic process,” and “macromolecule catabolic process” (Figure 4B; Supplementary Table S4). Interestingly, a large number of enriched GO terms related to catabolism and metabolism of host cell wall components, such as polysaccharides, xylan, and cellulose metabolism, were specifically upregulated in the resistant host. Although these genes related to cell wall degradation were significantly upregulated in the resistant host, the expression of these genes was lower than that in the susceptible host at an earlier stage (Figure 4B).

We also identified 489 significantly downregulated DEGs. Among them, 321 were specifically downregulated in Sumai3, only 93 were specifically downregulated in Fielder, while 75 were downregulated in both hosts (Figure 4C; Supplementary Table S4). DEGs specifically downregulated in Fielder were particularly associated with processes related to fatty acids and lipids, including biosynthetic process and metabolic process (Supplementary Table S4). We further identified processes particularly associated with the DEGs downregulated in both Fielder and Sumai3, including “transmembrane transport,” “localization,” and “amino acid transport” (Supplementary Table S4). Meanwhile, DEGs specifically downregulated in Sumai3 were particularly associated with “obsolete oxidation–reduction process,” “aldonic acid catabolic process,” and “response to cocaine” (Figure 4D; Supplementary Table S4). Additionally, we evaluated *F. graminearum* GO enrichments related to virulence and pathogenicity during infection of Sumai3, and observed decreases in the expression of *F. graminearum* genes involved in “response to toxic substance” and “response to fungicide” (Figure 4D). Taken together, these results highlighted the difference of *F. graminearum* infection between susceptible and resistant hosts.

To evaluate the effects of host resistance on the metabolic pathways of *F. graminearum*, KEGG pathway analyses were conducted with a *p*-value cutoff of <0.05. As shown in Supplementary Table S4, we observed that the DEGs specifically upregulated in Sumai3 were significantly associated with “starch and sucrose metabolism,” “isoflavonoid biosynthesis,” and “glutathione metabolism,” among others, while the DEGs specifically downregulated in Sumai3 were associated with “nitrogen metabolism,” “chloroalkane and chloroalkene degradation,” “ascorbate and aldarate metabolism,” and the metabolism of many other amino acids (Supplementary Table S4).

To determine the effect of the host on the expression pattern of *F. graminearum*, we further focused on the DEGs specifically downregulated in the resistant host. We first identified the DEGs that were upregulated in the susceptible host but downregulated in the resistant host. As shown in Figure 4E, 12 DEGs were identified as being upregulated in Fielder but downregulated in Sumai3 in the three comparisons, of which 7 DEGs were annotated to encode hypothetical proteins, while 5 DEGs were annotated to encode proteins with known functions, including “oxoglutarate iron-dependent oxygenase” (*FGSG_00048*), “major facilitator superfamily transporter” (*FGSG_02343*), “S-(hydroxymethyl) glutathione dehydrogenase” (*FGSG_10200*), “transporter MCH2” (*FGSG_03015*), and “intradiol ring-cleavage core” (*FGSG_03349*) (Supplementary Table S5). The GO terms “dioxygenase activity,” “transition metal ion binding,” and “oxidoreductase activity” were dominant in the main category of molecular function, while “organic hydroxy compound metabolic process” and “obsolete oxidation–reduction process” were significantly enriched in the main category of biological process (Supplementary Table S5). KEGG pathway analysis showed that *FGSG_00048* and *FGSG_03349* were significantly enriched in “Chlorocyclohexane and chlorobenzene degradation” (Supplementary Table S5). Only three DEGs were identified in the comparison of 36 HAI vs. 12 HAI (Supplementary Table S5). Based on the analysis of the potential function of these three DEGs, we found that *FGSG_06307* may be related to “N-acetyltransferase activity,” while *FGSG_03015* was involved in “Sphingolipid metabolism,” which is reasonable given that sphingolipids have been proven to play important roles in fungal growth and pathogenesis, and to act as receptors for some antifungal plant defensins (Supplementary Table S5) (Ramamoorthy et al., 2009). Thus, these DEGs specifically suppressed in the resistant host may represent direct targets of the plant defense against *F. graminearum* infection, which may also provide new targets for the drug control of FHB.

### DEGs related to pathogenicity and virulence were associated with different hosts

PHI-base (www.PHI-base.org) is a multispecies phenotype database of pathogen–host interactions, which is devoted to the identification and presentation of phenotypic information on pathogenicity and effector genes and their interactions with hosts (Urban et al., 2020). Here we applied our data to PHI-base to identify key genes related to pathogenicity and virulence. First, we curated the database of *F. graminearum* with the mutant phenotype, including 24 genes with “loss of pathogenicity,” 384 with “reduced virulence,” 4 with “increased virulence,” 999 with “unaffected virulence,” and 105 with “lethal” (Supplementary Table S6). When comparing these PHI-base genes with our transcriptomic data, we found that 732 PHI-base genes were expressed within 36 HAI, including 141 DEGs, of which 52 were related to “virulence” and “pathogenicity” (Supplementary Table S6).

**FIGURE 5:**
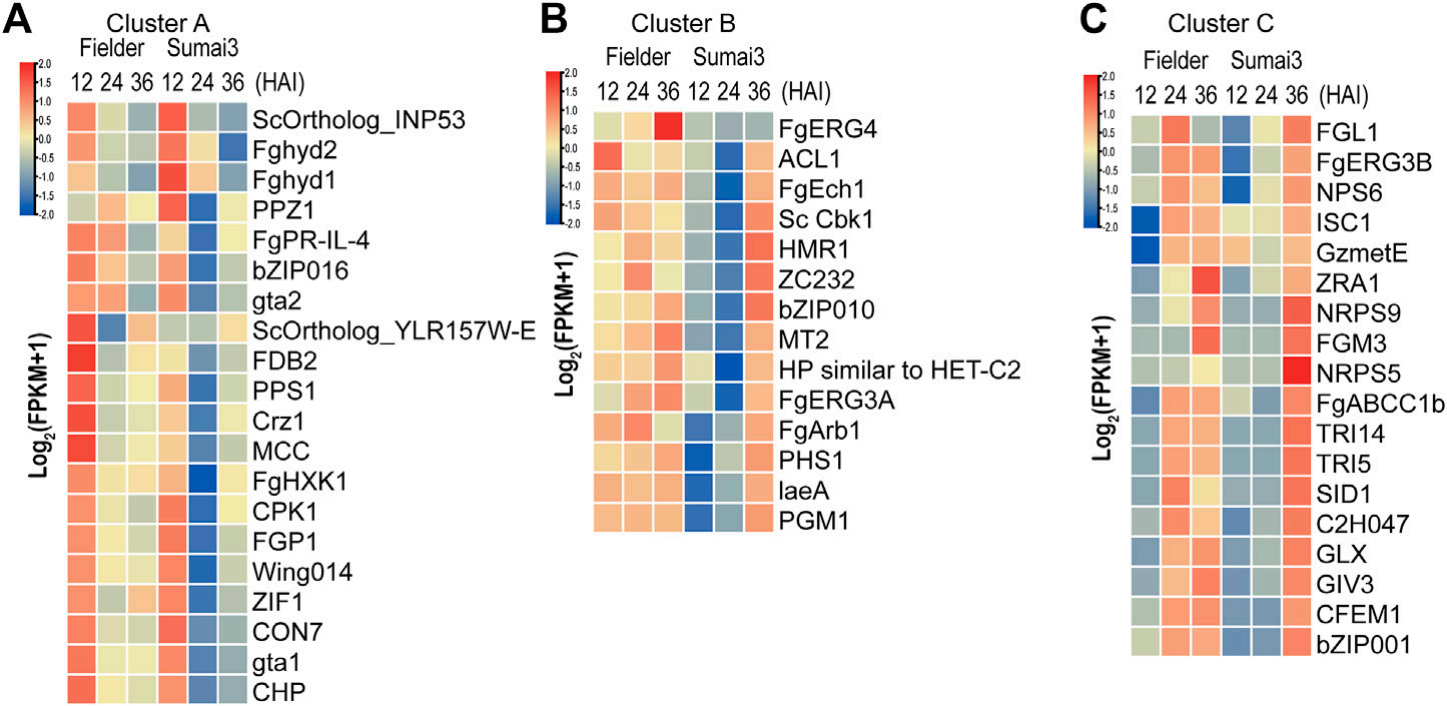
Identification of DEGs related to virulence and pathogenicity in PHI-base. **(A)** DEGs that exhibited high transcript abundance at 12 HAI and continuously decreased transcript abundance over the time course of infection. **(B)** DEGs that exhibited high transcript abundance at three timepoints after infection in Fielder, but did not exhibit high transcript abundance until 36 HAI in Sumai3. **(C)** DEGs that exhibited low transcript abundance at 12 HAI in both varieties and whose transcript abundance continuously increased over the time course of infection.

TBtools software was used to draw a heatmap of all 52 DEGs, and then cluster analysis of these DEGs was conducted. According to the difference of timing and expression patterns, these DEGs were classified into three groups: clusters A, B, and C (Figures 5A–C; Supplementary Table S6). Genes in cluster A were associated with extracellular signal transduction (*CPK1* and *FgHXK1*) and transcription factors (*CON7*, *bZIP016, Crz1*, and *ZIF1*), and their transcripts were highly abundant at 12 HAI in Fielder and Sumai3 and then decreased over time (Figure 5A). When compared with the levels in Fielder, DEGs in Sumai3 always exhibited lower transcript abundance in this cluster (Figure 5A). Among the 14 PHI-base DEGs in cluster B, *FgERG3A* and *FgERG4* were involved in steroid biosynthesis (Liu et al., 2013; Yun et al., 2014), while *PHS1* and *FgEch1* were involved in fatty acid elongation (Yun et al., 2015; Tang et al., 2020), and they showed high transcript abundance at three timepoints in Fielder, but only at 36 HAI in Sumai3 (Figure 5B). In cluster C, most DEGs in Fielder and Sumai3 exhibited low transcript abundance at 12 HAI and continuously increased over time (Figure 5C). This cluster included genes involved in DON biosynthesis (*TRI5* and *TRI14*) (Dyer et al., 2005; Alexander et al., 2009) and SMB (*NRPS5*, *NPS6,* and *NRPS9*) (Oide et al., 2006; Jia et al., 2019) (Supplementary Table S6). The differential expression patterns of these DEGs related to virulence and pathogenicity reveal the differences between the hosts with varying levels of resistance to the fungal pathogen.

### Dynamic expression of putative effectors in resistant and susceptible hosts

**FIGURE 6:**
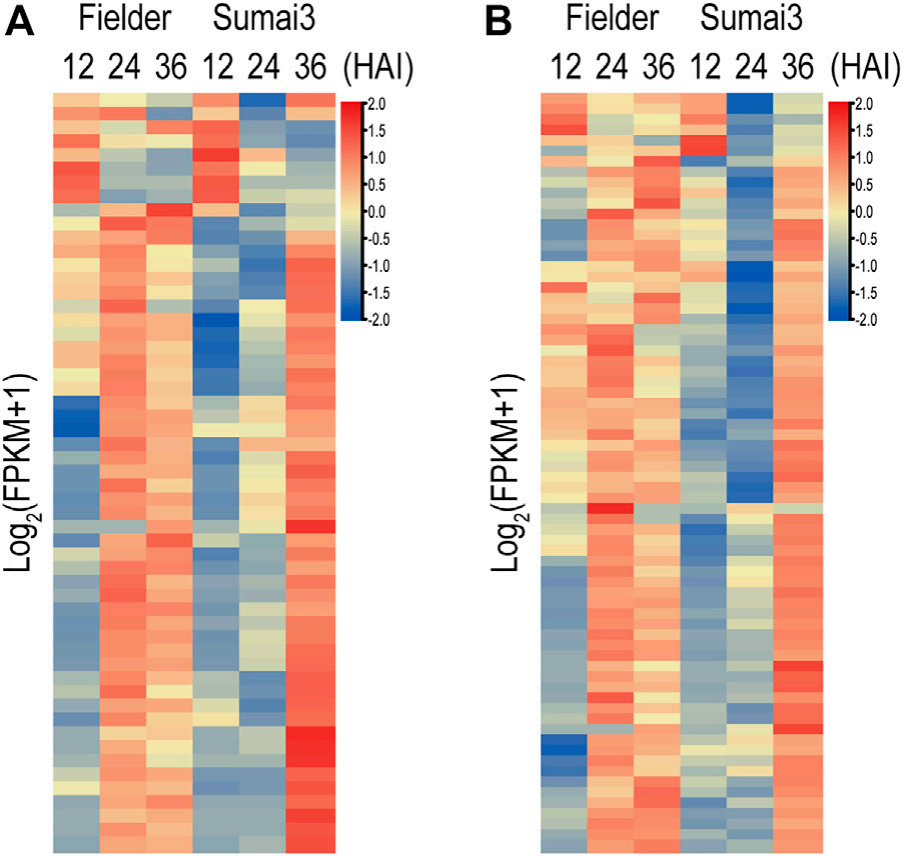
Putative fungal effectors during infection of Fielder and Sumai3 **(A)** Heatmap of 55 DEGs encoding putative effectors during infection of Fielder and Sumai3. **(B)** Evaluation of expression of 72 putative differentially expressed effectors in previous studies during infection of Fielder and Sumai3.

In the process of infecting plants, fungi secrete a range of effectors that contribute to their infection. In a previous study, a refined *F. graminearum* secretome of 574 proteins was predicted, 40% (231) of which were found in our RNA-Seq data (Brown et al., 2012) (Supplementary Table S7). Among the 231 genes encoding secreted proteins, 153 were differentially expressed in Fielder or Sumai3 within 36 HAI (Supplementary Table S7). EffectorP-fungi 3.0 software was subsequently used to determine whether these 153 encoded proteins were effectors, which finally identified 55 putative effectors (Figure 6A; Supplementary Table S7). Most of these putative effectors exhibited higher transcript abundance at 24 HAI in Fielder and at 36 HAI in Sumai3 (Figure 6A). Among them, 39 were described as apoplastic effectors, 4 as cytoplasmic effectors, 9 as apoplastic/cytoplasmic effectors, and 3 as cytoplasmic/apoplastic effectors (Supplementary Table S7). Three proteins, lipase FGL1 (FGSG_05906), phosphatase ISC1 (FGSG_03365), and hydrophobin Fghyd1 (FGSG_01763), were predicted being associated with reduced virulence in PHI-base (Supplementary Tables S7, S8) (Blumke et al., 2014; Yun et al., 2015; Quarantin et al., 2019). Twelve were predicted to be short peptides with 200 amino acids or less and cysteine content above 3%, among which FGSG_01763 (Fghyd1) has been proven to be a virulence gene (Supplementary Table S7). Further work is needed to determine whether the other 11 short peptides contribute to virulence. We also evaluated the expression of the previously reported putative effectors based on our data (Brown et al., 2017; Fabre et al., 2019; Miltenburg et al., 2022). Of all 236 expressed putative effectors, 72 were differentially expressed in Fielder or Sumai3, and 22 overlapped with our predictions (Figure 6B; Supplementary Table S7).

### Protein–protein interaction network reveals important biological activities in the early stage of infection

**FIGURE 7:**
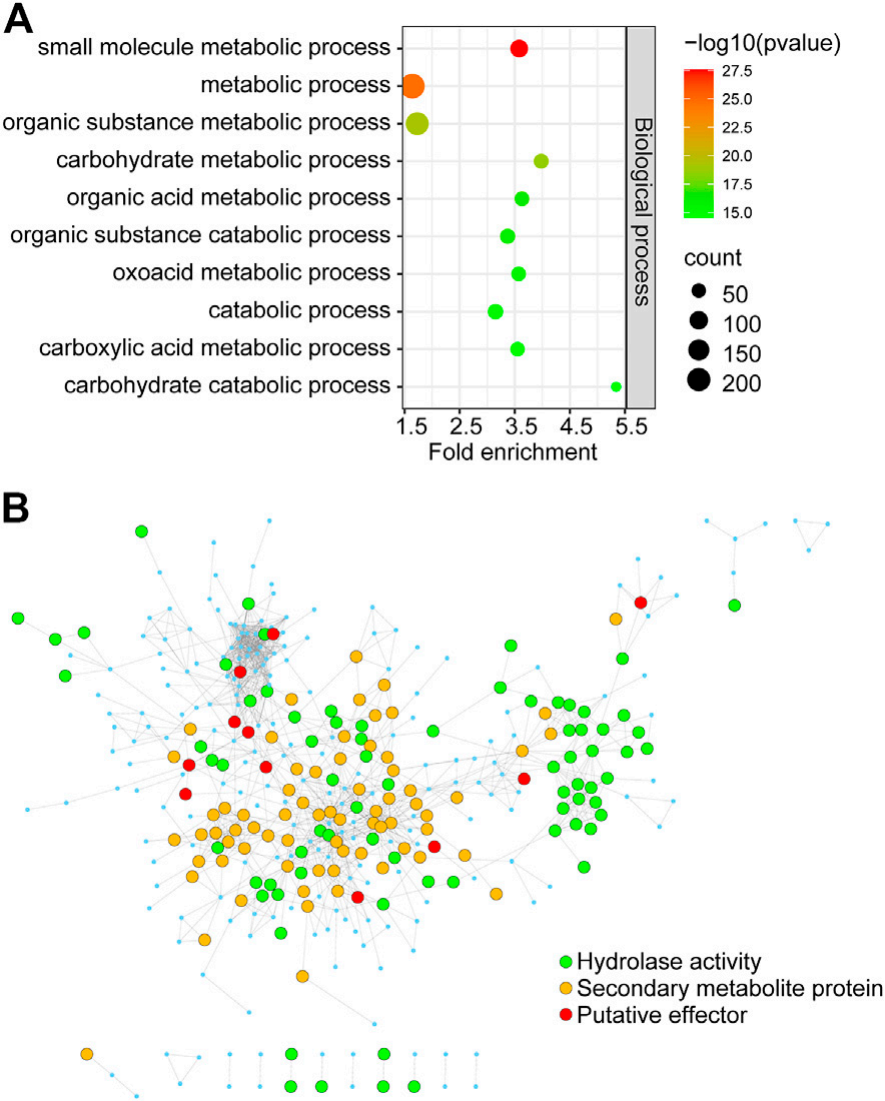
The PPI regulatory network of proteins encoded by *in planta Fusarium graminearum* DEGs **(A)** GO terms significantly associated with DEGs in the PPI regulatory network. **(B)** The *Fusarium graminearum* DEG regulatory network consists of 366 proteins. Each node represents a protein. Each line represents an interaction. Functions are color-coded. Green represents proteins involved in hydrolase activity, orange represents secondary metabolite proteins, and red represents putative effectors from this and previous studies.

To identify key genes from all of the DEGs during the course of infection, we constructed an *F. graminearum* protein–protein interaction (PPI) network. The online database String (Szklarczyk et al., 2015) was used to predict the PPI of proteins encoded by all 1,317 DEGs, and a network containing 366 proteins and 1,245 interaction events was identified (Supplementary Table S8). GO analysis revealed that these predicted DEGs were significantly associated with “carbohydrate metabolic process,” “organic substance metabolic process,” and “small-molecule metabolic process” (Figure 7A; Supplementary Table S8). The molecular function of 39 proteins in this network was annotated as “hydrolase activity,” including that of cellulase, xylanase, and pectinase, which may be involved in degradation of the host cell wall during infection (Figure 7B; Supplementary Table S8). KEGG analysis revealed the significant enrichment of “pyruvate metabolism,” “glycine, serine, and threonine metabolism,” and “galactose metabolism” (Supplementary Table S8). Eighty-two proteins were annotated to be involved in “biosynthesis of secondary metabolites,” including nine genes involved in “sterol biosynthetic process” (Figure 7B; Supplementary Table S8). We also found 28 putative effectors in this PPI network (Figure 7B; Supplementary Table S8), which may play important roles in invading host cells and overcoming plant immunity.

### Verification of the RNA-seq data by qRT-PCR

**FIGURE 8:**
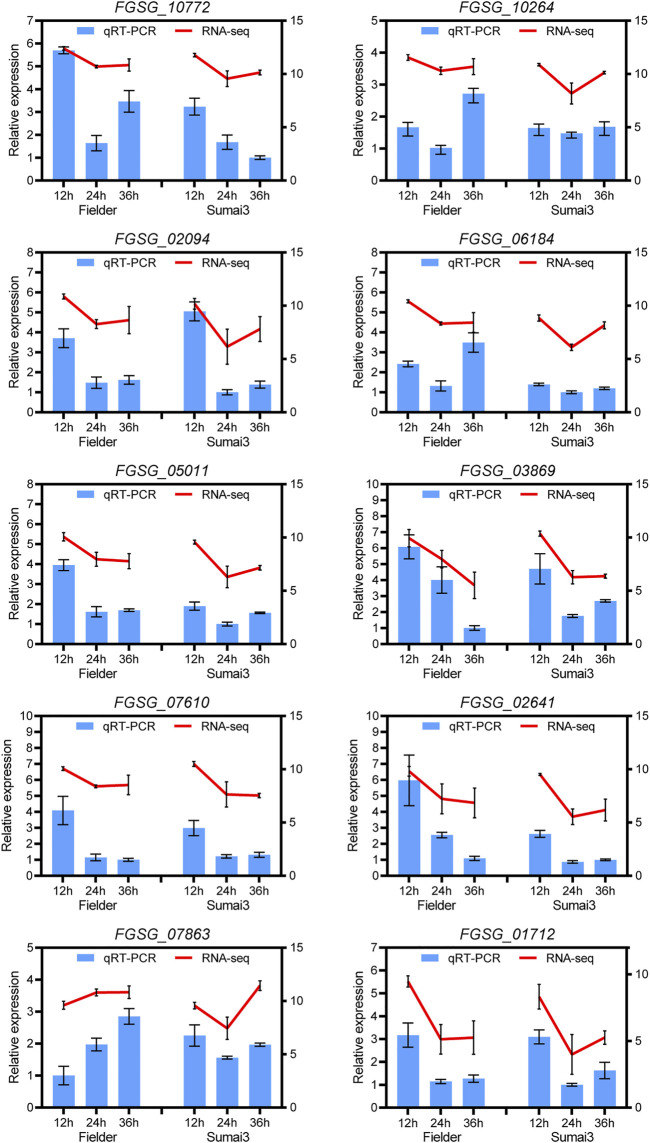
qRT-PCR analyses of ten randomly selected DEGs in Fielder and Sumai3. The left ordinate axis represents the relative expression level of DEGs by qRT-PCR, and the right ordinate axis represents the RNA-seq expression leve (log2(FPKM+1)). The *Fusarium graminearum* gene *FgEF1A* was used as internal reference and mean ± SD of data from three biological replicates was plotted.

To verify the reliability of the sequencing data, 10 DEGs in Fielder or Sumai3 were randomly selected and analyzed by qRT-PCR. As shown in Figure 8, most of the selected DEGs were consistent with the transcriptome data, which indicates that the RNA-SEQ results of this study are reliable.

## Discussion

FHB is a devastating fungal disease worldwide, which has a major impact on the sustainable production and food safety of cereals because of the lack of effective crop resistance and insensitivity to inherent fungicides of pathogens (Brown et al., 2017). Sumai3 is the most well-known FHB-resistant wheat variety, while Fielder is a wheat variety that is highly susceptible to FHB (Hofstad et al., 2016). Transcriptomic analysis is an effective way of studying plant–pathogen interactions (Kazan and Gardiner, 2018). In this study, we performed deep transcriptomic analyses on wheat spikes of Fielder and Sumai3, and identified and compared the *F. graminearum* gene expression profiles in susceptible and resistant wheat varieties. This transcriptomic study of *F. graminearum* genes *in planta* deepens our understanding of the differences in expression pattern of *F. graminearum* genes in susceptible and resistant wheat hosts, and reveals the effects of the specific molecular interactions between *F. graminearum* and different wheat varieties.

Through transcriptomic analysis, we identified a total of 6,106 genes expressed in Fielder or Sumai3 within 36 HAI. Most of the identified genes were expressed in both varieties. Of them, 1,317 were identified as DEGs at three timepoints during infection. GO analysis of the upregulated DEGs revealed great similarity of the enriched biological processes in both cultivars, mainly catabolism and metabolism. Moreover, GO analysis of the downregulated DEGs revealed that “transmembrane transport,” “localization,” and “acid metabolism” were terms that were significantly enriched in both varieties. We also observed some GO terms related to host immunity that were particularly associated with the DEGs downregulated in Sumai3, such as “response to toxic substance” and “response to fungicide” DEGs that were specifically upregulated in Sumai3 were particularly associated with “polysaccharide catabolic process,” “carbohydrate catabolic process,” and “macromolecule catabolic process,” and the significantly enriched KEGG pathways included “starch and sucrose metabolism,” “isoflavonoid biosynthesis,” and “glutathione metabolism”. Meanwhile, DEGs that were specifically downregulated in Sumai3 were particularly associated with “obsolete oxidation-reduction process,” “aldonic acid catabolic process,” and “response to cocaine,” and the significantly enriched KEGG pathway terms were “nitrogen metabolism,” “chloroalkane and chloroalkene degradation,” “ascorbate and aldarate metabolism,” and some other amino acid metabolism-related categories. Only 12 DEGs were identified as being upregulated in Fielder but downregulated in Sumai3 in the RNA-Seq comparisons. Of these, *FGSG_03015* was shown to be involved in “sphingolipid metabolism,” which had been proven to play important roles in fungal growth and pathogenesis (Ramamoorthy et al., 2009). Overall, our data highlight the difference in expression patterns of *F. graminearum* genes during infection of susceptible and resistant wheat hosts.

*Fusarium graminearum* genes closely related to pathogenicity and virulence are key to successful infection (Xu et al., 2022). Although some genes were previously described as being involved in pathogenicity or virulence, their patterns of expression during the infection of hosts with different levels of resistance remained unknown. PHI-base is a multispecies phenotype database for pathogen–host interactions, which integrates multiple genes related to virulence and pathogenicity (Urban et al., 2020). In this study, we compared our transcriptomic data with PHI-base and identified 53 DEGs with the mutant phenotype of “loss of pathogenicity” or “reduced virulence.” Compared with the levels in Sumai3, most of these DEGs exhibited higher transcript abundance in Fielder at one or more timepoints after infection. We also constructed an *F. graminearum* PPI network of all 1,317 DEGs. Our results revealed that, in the early stage of infection, “cell wall degradation,” “synthesis of secondary metabolites,” and “expression of virulence-related proteins” is dynamically regulated by hosts with different genetic backgrounds.

In the process of infection, pathogens secrete proteins named effectors into the plant apoplast or directly into plant cells to manipulate plant physiology and inhibit host defense responses (Gorash et al., 2021; Wang et al., 2022). *Fusarium graminearum* is a hemibiotroph combining certain features of biotrophic pathogens and necrotrophic ones and may possess a broad arsenal of effectors (Duba et al., 2018). The identification of pathogen effectors can contribute to the understanding of host–pathogen interactions, and may also provide a novel approach for crop resistance breeding (Gorash et al., 2021). For pathogens within the oomycete class, rapid detection of effectors by the presence of specific sequence motifs or “fingerprints” shared by effector families has speeded up the identification of resistance genes in potato (*Solanum tuberosum* L.) and *Arabidopsis* (Vleeshouwers et al., 2008; Vleeshouwers et al., 2011; Goritschnig et al., 2012). However, application of this method in identifying fungal pathogen effectors is still challenging because such specific sequence motifs or “fingerprints” are uncommon for effectors of fungal pathogens (Gorash et al., 2021). For *F. graminearum*, effectors are usually predicted using several criteria, such as whether or not they are secreted proteins, have a small size, or are enriched in cysteines (Gorash et al., 2021). In this study, we predicted 55 effectors from among the 1,317 DEGs, 22 of which were previously reported as putative effectors. Of these 55 putative effectors, 12 were predicted to be short peptides with 200 amino acids or less and a cysteine content above 3%, which are typical features of apoplastically accumulating effectors (Brown and Hammond-Kosak, 2015). Most of the predicted effectors were differentially expressed in Sumai3 and exhibited high transcript abundance at 36 HAI. As shown in previous studies, mutations of coding genes of three predicted effectors lead to reduced virulence, namely, a phosphatase gene (*FGSG_03365*) (Yun et al., 2015), a gene encoding Hydrophobin 1 (*FGSG_01763*) (Quarantin et al., 2019), and a gene encoding lipase (*FGSG_05906*) (Blumke et al., 2014). To date, few effectors have been demonstrated in *F. graminearum during* FHB infection in wheat (Miltenburg et al., 2022; Xu et al., 2022). Therefore, the 55 putative effectors identified in this study will help to mine new effectors and provide a reference for further functional studies of effectors.

In summary, we drew different gene expression profiles of *F. graminearum* in the process of infecting Fielder and Sumai3, and identified and compared the potential functions of these genes. Our results provide insights into the study of the interaction between FHB and susceptible/resistant wheat varieties, and also provide a large number of candidate genes which might be used as new targets for *F. graminearum* control and HIGS in wheat breeding for resistance to FHB in the future.

## Data Availability

The data presented in the study are deposited in the NCBI repository, accession number PRJNA909570.

## References

[B1] Alexander N. J. Proctor R. H. Mccormick S. P. (2009). Genes, gene clusters, and biosynthesis of trichothecenes and fumonisins in Fusarium. Toxin Rev. 28 (2-3), 198–215. 10.1080/15569540903092142

[B2] Bianchini A. Horsley R. Jack M. M. Kobielush B. Ryu D. Tittlemier S. (2015). DON occurrence in grains: A north American perspective. Cereal Food World 60 (1), 32–56. 10.1094/cfw-60-1-0032

[B3] Blumke A. Falter C. Herrfurth C. Sode B. Bode R. Schafer W. (2014). Secreted fungal effector lipase releases free fatty acids to inhibit innate immunity-related callose formation during wheat head infection. Plant Physiol. 165 (1), 346–358. 10.1104/pp.114.236737 24686113 PMC4012593

[B4] Boedi S. Berger H. Sieber C. Munsterkotter M. Maloku I. Warth B. (2016). Comparison of *Fusarium graminearum* transcriptomes on living or dead wheat differentiates substrate-responsive and defense-responsive genes. Front. Microbiol. 7, 1113. 10.3389/fmicb.2016.01113 27507961 PMC4960244

[B5] Boenisch M. J. Schafer W. (2011). *Fusarium graminearum* forms mycotoxin producing infection structures on wheat. BMC Plant Biol. 11 (1), 110–114. 10.1186/1471-2229-11-110 21798058 PMC3166921

[B6] Brown N. A. Antoniw J. Hammond-Kosack K. E. (2012). The predicted secretome of the plant pathogenic fungus Fusarium graminearum: A refined comparative analysis. PLoS One 7, e33731. 10.1371/journal.pone.0033731 22493673 PMC3320895

[B7] Brown N. A. Evans J. Mead A. Hammond-Kosack K. E. (2017). A spatial temporal analysis of the *Fusarium graminearum* transcriptome during symptomless and symptomatic wheat infection. Mol. Plant Pathol. 18 (9), 1295–1312. 10.1111/mpp.12564 28466509 PMC5697668

[B8] Brown N. A. Hammond-Kosack K. E. (2015). “Secreted biomolecules in fungal plant pathogenesis,” in Fungal bio-molecules: Sources, applications and recent developments (Chichester, UK: Wiley-Blackwell), 263–310.

[B9] Chen C. J. Chen H. Zhang Y. Thomas H. R. Frank M. H. He Y. H. (2020). TBtools: An integrative toolkit developed for interactive analyses of big biological data. Mol. Plant 13 (8), 1194–1202. 10.1016/j.molp.2020.06.009 32585190

[B10] Chetouhi C. Bonhomme L. Lasserre-Zuber P. Cambon F. Pelletier S. Renou J. P. (2016). Transcriptome dynamics of a susceptible wheat upon Fusarium head blight reveals that molecular responses to *Fusarium graminearum* infection fit over the grain development processes. Funct. Integr. Genomic. 16 (2), 183–201. 10.1007/s10142-016-0476-1 26797431

[B11] Cuomo C. A. Gueldener U. Xu J. R. Trail F. Turgeon B. G. Di Pietro A. (2007). The *Fusarium graminearum* genome reveals a link between localized polymorphism and pathogen specialization. Science 317 (5843), 1400–1402. 10.1126/science.1143708 17823352

[B12] Cuthbert P. A. Somers D. J. Thomas J. Cloutier S. Brule-Babel A. (2006). Fine mapping *Fhb1*, a major gene controlling Fusarium head blight resistance in bread wheat (*Triticum aestivum* L.). Theor. Appl. Genet. 112 (8), 1465–1472. 10.1007/s00122-006-0249-7 16518614

[B13] Duba A. Goriewa-Duba K. Wachowska U. (2018). A review of the interactions between wheat and wheat pathogens: Zymoseptoria tritici, Fusarium spp. and Parastagonospora nodorum. Int. J. Mol. Sci. 19, 1138. 10.3390/ijms19041138 29642627 PMC5979484

[B14] Dyer R. B. Plattner R. D. Kendra D. F. Brown D. W. (2005). *Fusarium graminearum TRI14* is required for high virulence and DON production on wheat but not for DON synthesis *in vitro* . J. Agric. Food Chem. 53 (23), 9281–9287. 10.1021/jf051441a 16277434

[B15] Fabre F. Vignassa M. Urbach S. Langin T. Bonhomme L. (2019). Time-resolved dissection of the molecular crosstalk driving Fusarium head blight in wheat provides new insights into host susceptibility determinism. Plant Cell Environ. 42 (7), 2291–2308. 10.1111/pce.13549 30866080

[B16] Figueroa M. Hammond-Kosack K. E. Solomon P. S. (2018). A review of wheat diseases-a field perspective. Mol. Plant Pathol. 19 (6), 1523–1536. 10.1111/mpp.12618 29045052 PMC6638159

[B17] Gorash A. Armoniene R. Kazan K. (2021). Can effectoromics and loss-of-susceptibility be exploited for improving Fusarium head blight resistance in wheat? Crop J. 9 (1), 1–16. 10.1016/j.cj.2020.06.012

[B18] Goritschnig S. Krasileva K. V. Dahlbeck D. Staskawicz B. J. (2012). Computational prediction and molecular characterization of an oomycete effector and the cognate arabidopsis resistance gene. Plos Genet. 8, e1002502. 10.1371/journal.pgen.1002502 22359513 PMC3280963

[B19] He Y. Wu L. Liu X. Jiang P. Yu L. Qiu J. (2020). TaUGT6, a novel UDP-Glycosyltransferase gene enhances the resistance to FHB and DON accumulation in wheat. Front. Plant Sci. 11, 574775. 10.3389/fpls.2020.574775 33178244 PMC7596251

[B20] Hofstad A. N. Nussbaumer T. Akhunov E. Shin S. Kugler K. G. Kistler H. C. (2016). Examining the transcriptional response in wheat *Fhb1* near-isogenic lines to *Fusarium graminearum* infection and deoxynivalenol treatment. Plant Genome 9, 1–15. 10.3835/plantgenome2015.05.0032 27898755

[B21] Jansen C. Von Wettstein D. Schafer W. Kogel K. H. Felk A. Maier F. J. (2005). Infection patterns in barley and wheat spikes inoculated with wild-type and trichodiene synthase gene disrupted Fusarium graminearum. Proc. Natl. Acad. Sci. U. S. A. 102 (46), 16892–16897. 10.1073/pnas.0508467102 16263921 PMC1283850

[B22] Ji F. Xu J. H. Liu X. Yin X. C. Shi J. R. (2014). Natural occurrence of deoxynivalenol and zearalenone in wheat from Jiangsu province, China. Food Chem. 157, 393–397. 10.1016/j.foodchem.2014.02.058 24679796

[B23] Jia L. J. Tang H. Y. Wang W. Q. Yuan T. L. Wei W. Q. Pang B. (2019). A linear nonribosomal octapeptide from *Fusarium graminearum* facilitates cell-to-cell invasion of wheat. Nat. Commun. 10, 922. 10.1038/s41467-019-08726-9 30804501 PMC6389888

[B24] Kang Z. Huang L. Buchenauer H. Han Q. Jiang X. (2004). Cytology of infection process of *Fusarium graminearum* on wheat spikes. Acta phytophy. Sin. 34 (4), 329–335.

[B25] Kazan K. Gardiner D. M. (2018). Transcriptomics of cereal-*Fusarium graminearum* interactions: What we have learned so far. Mol. Plant Pathol. 19 (3), 764–778. 10.1111/mpp.12561 28411402 PMC6638174

[B26] Khan M. K. Pandey A. Athar T. Choudhary S. Deval R. Gezgin S. (2020). Fusarium head blight in wheat: Contemporary status and molecular approaches. 3 Biotech. 10, 172. 10.1007/s13205-020-2158-x PMC7080935 32206506

[B27] Lewandowski S. M. Bushnell W. R. Evans C. K. (2006). Distribution of mycelial colonies and lesions in field-grown barley inoculated with Fusarium graminearum. Fusarium Graminearum. Phytopathol. 96 (6), 567–581. 10.1094/PHYTO-96-0567 18943174

[B28] Li G. Q. Zhou J. Y. Jia H. Y. Gao Z. X. Fan M. Luo Y. J. (2019). Mutation of a histidine-rich calcium-binding-protein gene in wheat confers resistance to Fusarium head blight. Nat. Genet. 51 (7), 1106–1112. 10.1038/s41588-019-0426-7 31182810

[B29] Lin F. Kong Z. X. Zhu H. L. Xue S. L. Wu J. Z. Tian D. G. (2004). Mapping QTL associated with resistance to Fusarium head blight in the Nanda2419 x Wangshuibai population. I. Type II resistance. Theor. Appl. Genet. 109 (7), 1504–1511. 10.1007/s00122-004-1772-z 15290053

[B30] Liu X. Jiang J. H. Yin Y. N. Ma Z. H. (2013). Involvement of FgERG4 in ergosterol biosynthesis, vegetative differentiation and virulence in *Fusarium graminearum* . Mol. Plant Pathol. 14, 71. 10.1111/j.1364-3703.2012.00829.x 22947191 PMC6638626

[B31] Macheleidt J. Mattern D. J. Fischer J. Netzker T. Weber J. Schroeckh V. (2016). Regulation and role of fungal secondary metabolites. Annu. Rev. Genet. 50, 371–392. 10.1146/annurev-genet-120215-035203 27732794

[B32] Mentges M. Glasenapp A. Boenisch M. Malz S. Henrissat B. Frandsen R. J. N. (2020). Infection cushions of *Fusarium graminearum* are fungal arsenals for wheat infection. Mol. Plant Pathol. 21 (8), 1070–1087. 10.1111/mpp.12960 32573086 PMC7368127

[B33] Miltenburg M. G. Bonner C. Hepworth S. Huang M. Rampitsch C. Subramaniam R. (2022). Proximity-dependent biotinylation identifies a suite of candidate effector proteins from Fusarium graminearum. Fusarium Graminearum. Plant J. 112 (2), 369–382. 10.1111/tpj.15949 35986640

[B34] Oide S. Moeder W. Krasnoff S. Gibson D. Haas H. Yoshioka K. (2006). NPS6, encoding a nonribosomal peptide synthetase involved in siderophore-mediated iron metabolism, is a conserved virulence determinant of plant pathogenic ascomycetes. Plant Cell 18 (10), 2836–2853. 10.1105/tpc.106.045633 17056706 PMC1626607

[B35] Puri K. D. Yan C. Leng Y. Zhong S. (2016). RNA-Seq revealed differences in transcriptomes between 3ADON and 15ADON populations of *Fusarium graminearum in vitro* and in planta. PLoS One 11, e0163803. 10.1371/journal.pone.0163803 27788144 PMC5082872

[B36] Quarantin A. Hadeler B. Kroger C. Schafer W. Favaron F. Sella L. (2019). Different hydrophobins of *Fusarium graminearum* are involved in hyphal growth, attachment, water-air interface penetration and plant infection. Front. Microbiol. 10, 751. 10.3389/fmicb.2019.00751 31031728 PMC6474331

[B37] Ramamoorthy V. Cahoon E. B. Thokala M. Kaur J. Li J. Shah D. M. (2009). Sphingolipid C-9 methyltransferases are important for growth and virulence but not for sensitivity to antifungal plant defensins in Fusarium graminearum. Eukaryot. Cell 8 (2), 217–229. 10.1128/EC.00255-08 19028992 PMC2643601

[B38] Savary S. Willocquet L. Pethybridge S. J. Esker P. Mcroberts N. Nelson A. (2019). The global burden of pathogens and pests on major food crops. Nat. Ecol. Evol. 3 (3), 430–439. 10.1038/s41559-018-0793-y 30718852

[B39] Scudamore K. A. (2008). Fate of fusarium mycotoxins in the cereal industry: Recent UK studies. World Mycotoxin J. 1 (3), 315–323. 10.3920/wmj2008.x034

[B40] Sieber C. M. Lee W. Wong P. Munsterkotter M. Mewes H. W. Schmeitzl C. (2014). The *Fusarium graminearum* genome reveals more secondary metabolite gene clusters and hints of horizontal gene transfer. PLoS One 9 (10), e110311. 10.1371/journal.pone.0110311 25333987 PMC4198257

[B41] Son H. Seo Y. S. Min K. Park A. R. Lee J. Jin J. M. (2011). A phenome-based functional analysis of transcription factors in the cereal head blight fungus, Fusarium graminearum. Fusarium Graminearum. Plos Pathog. 7, e1002310. 10.1371/journal.ppat.1002310 22028654 PMC3197617

[B42] Sperschneider J. Dodds P. N. (2022). EffectorP 3.0: Prediction of apoplastic and cytoplasmic effectors in fungi and oomycetes. Mol. Plant Microbe. Interact. 35 (2), 146–156. 10.1094/MPMI-08-21-0201-R 34698534

[B43] Su Z. Q. Bernardo A. Tian B. Chen H. Wang S. Ma H. X. (2019). A deletion mutation in TaHRC confers Fhb1 resistance to Fusarium head blight in wheat. Nat. Genet. 51 (7), 1099–1105. 10.1038/s41588-019-0425-8 31182809

[B44] Szklarczyk D. Franceschini A. Wyder S. Forslund K. Heller D. Huerta-Cepas J. (2015). STRING v10: Protein-protein interaction networks, integrated over the tree of life. Nucleic Acids Res. 43 (1), D447–D452. 10.1093/nar/gku1003 25352553 PMC4383874

[B45] Tang L. Yu X. Y. Zhang L. Zhang L. Y. Chen L. Zou S. S. (2020). Mitochondrial FgEch1 is responsible for conidiation and full virulence in Fusarium graminearum. Curr. Genet. 66 (2), 361–371. 10.1007/s00294-019-01028-z 31463774

[B46] Trail F. (2009). For blighted waves of grain: *Fusarium graminearum* in the postgenomics era. Plant Physiol. 149 (1), 103–110. 10.1104/pp.108.129684 19126701 PMC2613717

[B47] Urban M. Cuzick A. Seager J. Wood V. Rutherford K. Venkatesh S. Y. (2020). PHI-Base: The pathogen-host interactions database. Nucleic Acids Res. 48 (D1), D613–D620. 10.1093/nar/gkz904 31733065 PMC7145647

[B48] Vleeshouwers V. G. A. Rietman H. Krenek P. Champouret N. Young C. Oh S. K. (2008). Effector genomics accelerates discovery and functional profiling of potato disease resistance and phytophthora infestans avirulence genes. PLoS One 3, e2875. 10.1371/journal.pone.0002875 18682852 PMC2483939

[B49] Vleeshouwers V. G. A. A. Raffaele S. Vossen J. H. Champouret N. Oliva R. Segretin M. E. (2011). Understanding and exploiting late blight resistance in the age of effectors. Annu. Rev. Phytopathol. 49, 507–531. 10.1146/annurev-phyto-072910-095326 21663437

[B50] Walter S. Nicholson P. Doohan F. M. (2010). Action and reaction of host and pathogen during Fusarium head blight disease. New Phytol. 185 (1), 54–66. 10.1111/j.1469-8137.2009.03041.x 19807873

[B51] Wang Y. Pruitt R. N. Nurnberger T. Wang Y. C. (2022). Evasion of plant immunity by microbial pathogens. Nat. Rev. Microbiol. 20, 449–464. 10.1038/s41579-022-00710-3 35296800

[B52] Xu M. Wang Q. H. Wang G. H. Zhang X. Liu H. Q. Jiang C. (2022). Combatting Fusarium head blight: Advances in molecular interactions between Fusarium graminearum and wheat. Phytopathology Res. 4, 37. 10.1186/s42483-022-00142-0

[B53] Yang G. Pan Y. Zhao Q. L. Huang J. Q. Pan W. Q. Cui L. C. (2022). Genome-wide identification and characterization of rna/dna differences associated with *Fusarium graminearum* infection in wheat. Int. J. Mol. Sci. 23, 7982. 10.3390/ijms23147982 35887327 PMC9316857

[B54] Yin J. Han X. Zhu Y. Fang Z. Gao D. Ma D. (2022). Transcriptome profiles of circular rnas in common wheat during fusarium head blight disease. Data 7 (9), 121. 10.3390/data7090121

[B55] Yun Y. Z. Liu Z. Y. Yin Y. N. Jiang J. H. Chen Y. Xu J. R. (2015). Functional analysis of the *Fusarium graminearum* phosphatome. New Phytol. 207 (1), 119–134. 10.1111/nph.13374 25758923

[B56] Yun Y. Z. Yin D. F. Dawood D. H. Liu X. Chen Y. Ma Z. H. (2014). Functional characterization of FgERG3 and FgERG5 associated with ergosterol biosynthesis, vegetative differentiation and virulence of Fusarium graminearum. Fusarium Graminearum. Fungal Genet. Biol. 68, 60–70. 10.1016/j.fgb.2014.04.010 24785759

[B57] Zhou J. M. Zhang Y. L. (2020). Plant immunity: Danger perception and signaling. Cell 181 (5), 978–989. 10.1016/j.cell.2020.04.028 32442407

[B58] Zhu G. R. Gao C. Y. Wu C. Y. Li M. Xu J. R. Liu H. Q. (2021). Comparative transcriptome analysis reveals distinct gene expression profiles in *Brachypodium distachyon* infected by two fungal pathogens. BMC Plant Biol. 21, 304. 10.1186/s12870-021-03019-0 34193039 PMC8243454

