# Supplementary material for: Comparative transcriptomics identifies the key in planta-expressed genes of Fusarium graminearum during infection of wheat varieties
Source: Front Genet. 2023 Apr 18;14:1166832. doi: 10.3389/fgene.2023.1166832 (PMC10151574; doi:10.3389/fgene.2023.1166832)
Supplement: Supplementary file 3 [file Table1.DOCX]

Supplementary Material

Comparative Transcriptomics Identifies the Key In Planta-Expressed Genes of Fusarium graminearum during Infection of Wheat Varieties

Qiang Tu* et al.,

*** Correspondence:**  Gang Li: gang.li@njau.edu.cn; Yi He: yihe@jaas.ac.cn

# Supplementary Figures and Tables


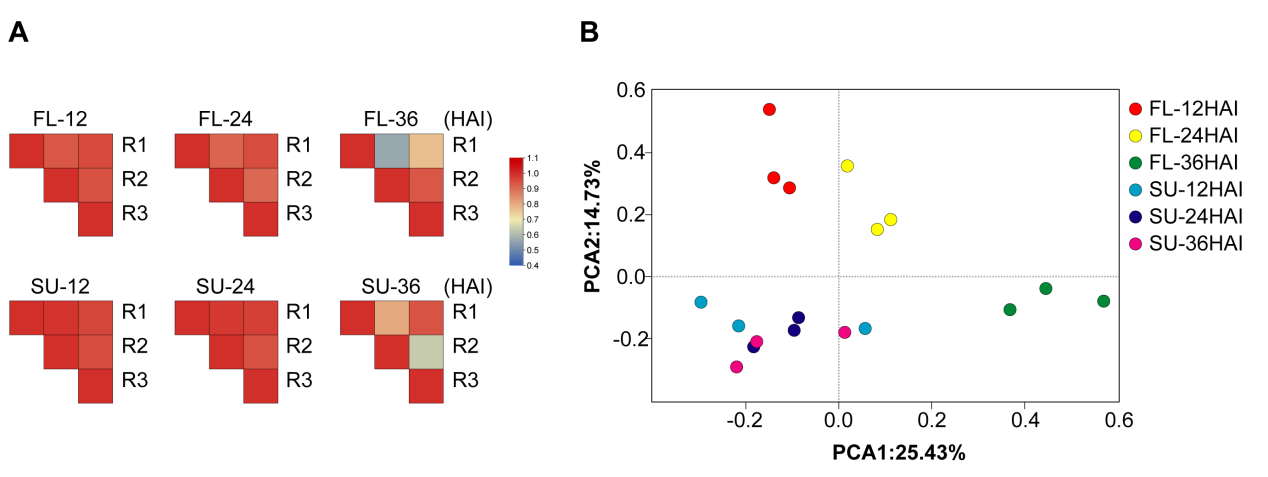


**Supplementary Figure S1.** Statistical analysis of all expressed genes. (A) Pearson’s correlation coefficients of three biological replicates for each condition. The reads generated by RNA-Seq were converted into FKPM values and were then used for this analysis. (B) Principal component analysis of *F. graminearum* genes identified in RNA-Seq analysis.

**Supplementary Table S1.** Primer sequences used in this study.

**Supplementary Table S2.**  Lists of F. graminearum genes and their expression in planta. This table included genes mapped to the F. graminearum PH-1 genome, correlation analysis, principal component analysis, planta-expressed genes and differentially expressed genes. FL, Fielder; SU, Sumai3.

**Supplementary Table S3.** Lists of upregulated and downregulated F. graminearum DEGs in Fielder and Sumai3, and their GO enrichment analysis.

**Supplementary Table S4.** GO and KEGG enrichment analyses of F. graminearum DEGs in Fielder and Sumai3.

**Supplementary Table S5.** GO and KEGG enrichment analyses of F. graminearum DEGs that were upregulated in the susceptible host but downregulated in the resistant host.

**Supplementary Table S6.** List of DEGs related to pathogenicity and virulence by comparing with the PHI-base, DEGs with mutant phenotypes of “Loss of pathogenicity” and “Reduced virulence” were paid more attention.

**Supplementary Table S7.** List of effectors predicted by EffectorP-fungi 3.0 software.

**Supplementary Table S8.** List of DEGs in the protein–protein interaction network, and corresponding GO enrichment analysis and KEGG enrichment analysis.
